# Insight into Rice Resistance to the Brown Planthopper: Gene Cloning, Functional Analysis, and Breeding Applications

**DOI:** 10.3390/ijms252413397

**Published:** 2024-12-13

**Authors:** Yangdong Ye, Shangye Xiong, Xin Guan, Tianxin Tang, Zhihong Zhu, Xiao Zhu, Jie Hu, Jianguo Wu, Shuai Zhang

**Affiliations:** State Key Laboratory for Ecological Pest Control of Fujian and Taiwan Crops, College of Plant Protection, Fujian Agriculture and Forestry University, Fuzhou 350002, China; yeyangdong6246@163.com (Y.Y.); xsy_986023@163.com (S.X.); guanxin001027@163.com (X.G.); 15201556632@163.com (T.T.); zhuzhihong20021122@163.com (Z.Z.); zxnfhg@163.com (X.Z.); jackhujie1526@163.com (J.H.)

**Keywords:** rice, brown planthopper, resistance genes, salivary proteins, insect-resistant breeding

## Abstract

This review provides a comprehensive overview of the current understanding of rice resistance to the brown planthopper (BPH), a major pest that poses significant threats to rice production through direct feeding damage and by transmitting viruses such as Rice grassy stunt virus (RGSV) and Rice ragged stunt virus (RRSV). We highlight the emergence of various BPH biotypes that have overcome specific resistance genes in rice. Advances in genetic mapping and cloning have identified 17 BPH resistance genes, classified into typical R genes encoding nucleotide-binding leucine-rich repeat (NLR) proteins and atypical R genes such as lectin receptor kinases and proteins affecting cell wall composition. The molecular mechanisms of these genes involve the activation of plant defense pathways mediated by phytohormones like jasmonic acid (JA), salicylic acid (SA), and ethylene, as well as the production of defensive metabolites. We also examine the complex interactions between BPH salivary proteins and rice defense responses, noting how salivary effectors can both suppress and trigger plant immunity. The development and improvement of BPH-resistant rice varieties through conventional breeding and molecular marker-assisted selection are discussed, including strategies like gene pyramiding to enhance resistance durability. Finally, we outline the challenges and future directions in breeding for durable BPH resistance, emphasizing the need for continued research on resistance mechanisms and the development of rice varieties with broad-spectrum and long-lasting resistance.

## 1. Introduction

Rice is one of the most important staple crops in China. Statistics indicate that the annual average rice yield in China reaches 211.474 million tons, with a per capita possession of 152 kg [1]. Given that over 60% of the Chinese population relies on rice as their staple food, rice production is crucial for national food security. Therefore, the stability of rice yield is vital for the national economy and the well-being of the people [2,3,4]. The brown planthopper (*Nilaparvata lugens* Stål, BPH), a major pest of rice, has the capability of long-distance migration and poses a serious threat to rice production in China and other major rice-producing countries such as Vietnam, the Philippines, Thailand, and Indonesia, sometimes even causing severe “hopper burn” [5]. According to the “China Agricultural Yearbook”, BPH affects over 25 million hectares annually, resulting in an approximate loss of 3 million tons of rice [6]. Additionally, BPH is the principal vector for Rice grassy stunt virus (RGSV) and Rice ragged stunt virus (RRSV) [7,8,9,10]. These viruses are transmitted by BPH feeding, spreading to the phloem of rice plants, which leads to stunted growth, increased tillering, and reduced fertility, sometimes resulting in complete crop failure [11,12]. Currently, controlling BPH primarily relies on chemical pesticides [13]. However, this approach is costly, environmentally damaging, and potentially harmful to non-target organisms, while also contributing to increased resistance in planthoppers [14,15,16,17]. Consequently, researching and developing new rice varieties resistant to BPH has become a cost-effective, environmentally friendly, and ecologically safe control strategy [18,19,20,21].

Previous studies have reviewed the origins, mapping, and cloning of BPH resistance genes, along with the associated mechanisms [5,22,23,24,25,26]. In contrast, this review advances the field by providing a comprehensive analysis of BPH biotypes and their resistance identification methods. We delve into the mapping and cloning of resistance genes and quantitative trait loci (QTLs) in rice, as well as the molecular mechanisms underlying rice resistance to BPH. A key innovation of this review is the exploration of how BPH salivary proteins regulate interactions between rice and the planthopper, offering new insights into the molecular dialogue between host and pest. Additionally, we discuss research on viruses transmitted by BPH, such as RGSV and RRSV, highlighting how these viruses impact rice physiology and how resistance mechanisms against BPH also intersect with viral defense pathways. We also examine the practical applications of these findings in rice breeding, proposing strategies to identify and utilize target genes for developing new rice varieties resistant to both BPH and BPH-transmitted viruses. By deeply analyzing the interaction mechanisms between rice and BPH, including the aspect of virus transmission, this review aims to provide a sustainable molecular design approach for rice breeding, promoting agricultural development.

## 2. Adaptation of BPH Biotypes to Rice Resistance Genes

During the extended process of coevolution, rice has evolved a variety of resistance genes to combat the invasion of BPH, which, in turn, have gradually evolved different populations capable of overcoming specific resistance genes, known as biotypes or virulent types. Currently, there are at least ten recognized BPH biotypes, including biotype I, biotype II, biotype III, Coimbatore, Hyderabad, Parwanipur from Nepal, Pantnagar, Mekong Delta from Vietnam and Australia, and Mindanao from the Philippines [27,28,29]. Among these, biotype I exhibits the weakest virulence towards rice, whereas biotypes II and III display stronger pathogenicity and have overcome a broader range of rice varieties. Consequently, in response to the threat posed by biotypes II and III, rice has evolved a greater number of SNP sites to counteract their attacks [30]. Using RAPD-PCR technology, researchers analyzed the genomic DNA polymorphism of BPH populations (long-winged and short-winged types) collected from various locations and times, revealing significant genetic differences among these populations. Moreover, even within the same virulence group, RAPD polymorphism varied among individual insects. Further studies demonstrated clear genetic differentiation between populations of different virulence, and the genetic characteristics of BPH virulence were found to be potentially linked to sex chromosomes, with each virulent population exhibiting unique genetic bands [31]. Additionally, significant differences were observed in the virulence phenotypes, genetic backgrounds, and gene expression levels among different BPH biotypes. Alanine transaminase (ALT) plays a crucial role in the adaptation of BPH populations to the resistant rice variety IR36 by mediating the conversion of alanine to pyruvate, a key process for downstream energy resource allocation. Increased ALT activity provides additional energy reserves for BPH populations adapting to IR36, thereby enhancing their detoxification capacity and ecological adaptability against the resistance traits of IR36. This pathway demonstrates conservation across BPH populations adapting to rice varieties with various resistance genes [32]. Research conducted by the International Rice Research Institute (IRRI) has demonstrated that cultivating rice varieties with different resistance genes can effectively control the growth of BPH populations and, to some extent, slow the rate of variation in their biotypes or virulence, thereby achieving sustainable resistance [33].

## 3. Genetic Mapping and Cloning Progress of Rice BPH Resistance Genes

Since the Green Revolution in rice that began in the 1960s, scientists from Japan, India, and China have progressively conducted genetic mapping studies for rice resistance genes against BPH [34]. In 1969, the International Rice Research Institute first screened and identified the resistant indica rice variety Mudgo [35]. To date, researchers have identified a vast array of insect-resistant germplasm resources within cultivated and wild rice and have localized 80 resistance genes/QTLs against BPH (Table S1) [24,25,36]. These genes or QTLs are primarily clustered on chromosomes 3, 4, 6, and 12. In different regions of the chromosome, the insect resistance traits expressed by various genes may exhibit subtle differences, which could be due to these genes being tightly linked, allelic, or even different expressions of the same gene. These clustered genes provide a rich resource for the development of new rice varieties resistant to BPH. Utilizing existing molecular markers, it is possible to effectively pyramid multiple resistance genes, thereby enhancing the insect resistance of rice. This has significant implications for improving the sustainable production of crops.

**Figure 1:**
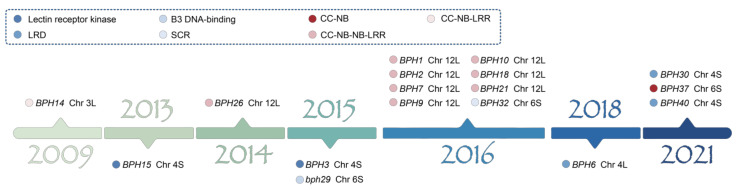
Timeline of cloned genes for brown planthopper resistance in rice. This figure illustrates a timeline of cloned rice genes conferring resistance to BPH, spanning the years 2009 to 2021. Gene symbols are classified based on their protein domains, including lectin receptor kinases, LRD, B3 DNA-binding, CC-NB, CC-NB-LRR, and CC-NB-NB-LRR, indicating diverse mechanisms of resistance. The chromosome positions (e.g., Chr 3L, Chr 12L) highlight the genomic locations of these genes. Each gene is marked with a unique color based on its year of discovery, facilitating a visual overview of the progress in rice resistance breeding against BPH.

To date, 17 resistance genes against BPH have been successfully cloned in rice (Figure 1, Table S2). Based on the amino acid domains they encode, these can be categorized into two major types: R genes and non-R genes. Typical R genes encode nucleotide-binding leucine-rich repeat (NLR) proteins, which are intracellular immune receptors. These proteins typically recognize pathogen effector molecules directly, activate immune responses, and often trigger localized cell death to limit pathogen spread. Atypical R genes encode proteins that do not conform to the classical NLR structure, including receptor-like kinases (RLKs) and receptor-like proteins (RLPs). These proteins are usually localized to the cell membrane and are responsible for detecting extracellular signals. Additionally, some non-canonical resistance proteins function by modifying cell wall composition or indirectly regulating signaling pathways. Both types of genes play essential roles in plant immunity by recognizing pathogen signals and triggering defense responses. Some atypical R genes can collaborate with typical R genes, amplifying immune responses through interconnected signaling pathways and enhancing the overall resistance of the plant [37]. For instance, the *Bph14* gene, originally identified in the resistant rice line B5 derived from the wild rice *Oryza officinalis*, encodes a protein with a CC-NB-LRR (coiled-coil–nucleotide binding site–leucine-rich repeat) structure, representing a typical R gene [38]. *Bph15* and *Bph3* are allelic, encoding lectin receptor kinase proteins, and belong to the non-R gene category [39,40].

### 3.1. BPH Resistance Genes That Are Classified as Typical R Genes

Plant resistance (R) genes recognize pathogen-specific effector molecules and initiate robust immune responses. These responses often lead to programmed cell death at the infection site, effectively preventing the spread of the pathogen. As a typical R gene, *Bph14* encodes a CC-NB-LRR protein that activates the salicylic acid (SA) signaling pathway and induces the expression of defense-related genes. Studies have shown that BPH14 interacts with the transcription factors WRKY46 and WRKY72, promoting their protein accumulation and transcriptional activation activity, which regulates downstream defense genes associated with callose synthesis and cytoplasmic kinases, thereby conferring effective resistance to BPH [41]. Recent research further revealed that in susceptible plants, the BPH14-Interacting Salivary Protein (BISP) targets the rice receptor-like cytoplasmic kinase *Os*RLCK185, suppressing basal defense responses. In resistant plants, the rice immune receptor BPH14 directly recognizes and binds to BISP, thereby activating host plant resistance (HPR). Additionally, BISP, BPH14, and the selective autophagy cargo receptor *Os*NBR1 interact, with *Os*NBR1 delivering BISP to *Os*ATG8 for degradation. Through autophagy-mediated regulation of BISP levels, plants restore cellular homeostasis after brown planthopper feeding ceases, reducing HPR activity and mitigating the negative impacts of overactive immunity on plant growth and productivity [42]. This study uncovers a “three-way interaction” mechanism involving an insect salivary protein, a plant immune receptor, and autophagy regulation, offering new insights for developing high-yield and insect-resistant crops.

The *Bph9* gene in rice, originally identified in the traditional Sri Lankan rice varieties Pokkali and Kaharamana, encodes a protein essential for plant immunity, comprising CC (coiled-coil), NBS (nucleotide-binding site), and LRR (leucine-rich repeat) domains [43]. The CC domain, particularly amino acids 97–115, is crucial for protein self-interaction and activating downstream signals. Bph9 contains two NBS domains: NBS1, which has a diminished regulatory role due to the absence of the ARC2 sequence, and NBS2, which fully inhibits CC domain activation, functioning as a negative regulator. The LRR domain counteracts NBS2’s inhibition, enhancing signal transduction by recognizing pathogen signals and modulating internal signaling to maintain immune balance. High expression of *Bph9* in vascular tissue induces hypersensitive necrosis and activates the SA and jasmonic acid (JA)-dependent resistance pathways. *Bph9* exhibits significant sequence diversity within rice germplasm, forming a complex resistance locus alongside genes like *Bph1*, *Bph2*, *Bph7*, *Bph9*, *Bph10*, *Bph18*, *Bph21*, and *Bph26*. This genetic diversity leads to various allelic types, providing differential resistance to BPH biotypes and representing a vital evolutionary strategy for rice to combat BPH infestations.

*Bph37* encodes a protein that includes a CC-NB domain but lacks an LRR domain. This gene was cloned within the 1.20–1.57 Mb region on the short arm of chromosome 6 in the cultivated rice SE382, following genomic analysis and genome-wide association studies of 1520 rice germplasms [30]. In rice materials resistant to BPH, an insertion mutation occurs in the second exon of the *Bph37* gene, leading to premature termination of the encoded amino acid sequence and the absence of the LRR domain. This structural variation may be related to the gene’s insect resistance mechanism. In contrast, rice varieties susceptible to BPH, such as Nipponbare and Kasalath, retain a complete CC-NB-LRR structure.

### 3.2. BPH Resistance Genes That Are Classified as Atypical R Genes

*Bph30* and *Bph40* belong to the LRR domain gene family and play crucial roles in rice resistance to BPH by strengthening cell walls [44]. *Bph30*, cloned from the traditional variety AC-1613, encode a protein with two LRD domains localized to the endoplasmic reticulum, vacuolar membrane, and extracellular vesicles. It is highly expressed in sclerenchyma cells, promoting the synthesis of cellulose and hemicellulose, which thickens cell walls and forms a barrier that prevents BPH stylet penetration and sap feeding. Similarly, *Bph40* was identified from the indica varieties SE232, SE67, and C334 through genome-wide association and homologous gene analysis. It encodes an LRD family protein expressed in the cell walls of leaf sheaths, significantly enhancing the expression of cell wall-associated genes and increasing cellulose and hemicellulose content. Together, *Bph30* and *Bph40* improve structural defenses, effectively protecting rice plants from BPH infestations [44].

*Bph6* is a significant rice resistance gene against planthoppers, encoding a lectin receptor-like kinase protein localized in extracellular vesicles. Originating from the cultivated variety Swarnalata, *Bph6* is positioned between molecular markers H and Y9 on chromosome 4’s long arm [45]. BPH6 interacts with the extracellular vesicle subunit *Os*EXO70E1, promoting exocytosis and enhancing cell wall stability and thickness. This fortified cell wall impedes BPH and white-backed planthopper stylet penetration. Additionally, *Bph6* activates the coordinated cytokinin, SA, and JA signaling pathways, providing broad-spectrum resistance without yield loss [45]. Further studies show that *Os*EXO70H3, another EXO70 family member, interacts with BPH6 and the S-adenosylmethionine synthetase-like protein (SAMSL). This interaction increases SAMSL secretion, enhancing lignin deposition in cell walls and strengthening insect resistance. Deficiencies in *Os*EXO70H3 or SAMSL reduce lignin content, diminishing planthopper resistance [46].

### 3.3. BPH Resistance Genes That Are Not Classified as R Genes

*Bph15* and *Bph3* are genes that encode rice lectin receptor kinases (*Os*LecRKs), located on chromosome 4 of rice [39,40]. These genes belong to the family of plant receptor kinases and play a critical role in biological defense. The *Bph15* gene is located on the short arm of chromosome 4 in *O. officinalis*, within the 6.68–6.90 Mb region between RG1 and RG2, and exhibits resistance to BPH biotypes 1, 2, and 3 [5]. *Bph15* not only participates in insect defense but also affects rice growth and development through the function of its kinase domain. Studies have found that *Bph15* promotes the expression of the α-amylase gene, enhancing seed vigor and subsequently increasing germination rates. Additionally, BPH15 enhances resistance to BPH by interacting with actin depolymerizing factor (ADF) to activate the expression of defense genes in rice [39].

The *Bph3* gene consists of three tandemly repeated lectin receptor kinase genes (*OsLecRK1*-*OsLecRK3*), located within a 79 kb segment on the short arm of chromosome 4 in the Sri Lankan variety *Rathu Heenati* [40]. These three genes exhibit an additive effect, where *OsLecRK1* contributes approximately 50% of the effect, while *OsLecRK2* and *OsLecRK3* each contribute around 25%. *BPH3* not only confers resistance to BPH but also to the white-backed planthopper, displaying broad-spectrum insect resistance. This gene is primarily localized to the plasma membrane and participates in regulating rice cell signal transduction through its kinase activity, enhancing the plant’s overall insect resistance [40]. *bph29* is the only known cloned recessive gene for resistance to BPH, encoding a resistance protein with a B3 DNA-binding domain. This gene is specifically located within a 24kb segment on the short arm of chromosome 6 in the introgression line RBPH54 of the common wild rice *O. rufipogon* [47]. *bph29* is expressed specifically in vascular tissues, and its mechanism includes activating the SA signaling pathway and suppressing the JA/ethylene signaling pathway. Such regulatory modulations help the plant initiate specific defense responses during insect attacks. *Bph32* encodes a short homologous repeat sequence (SCR) protein localized to the plasma membrane, cloned from a 190 kb segment between markers RM19291 and RM8072 on the short arm of chromosome 6 in Ptb33 [48]. This gene is highly expressed in the leaf sheath and primarily mediates resistance to BPH by inhibiting its feeding behavior, thereby blocking the pest’s nutrient intake pathways and increasing the crop’s survival chances.

## 4. BPH Resistance-Related Genes and Mechanisms

In natural environments, rice frequently suffers from BPH attacks, prompting the evolution of a series of complex defense mechanisms. To date, 40 BPH resistance-related genes have been identified mainly through reverse genetics, encoding proteins such as kinases, hormone signaling components, and transcription factors (Table 1). The mitogen-activated protein kinase (MAPK) signaling cascade, involving kinases like *Os*MPK1, *Os*MPK3, and *Os*MPK4, is pivotal in rice’s immune response to BPH [49,50,51,52]. Plant hormones, particularly JA, SA, and ethylene (ET), play critical roles in defending against herbivorous insect invasions, with the JA signaling pathway playing a central role in regulating plant resistance to herbivores [24]. Key transcription factors, including b-ZIP, *Os*SPL10, NAC, and MYB, regulate defense gene expression across multiple pathways [53,54,55,56,57,58]. Additionally, miRNAs like *OsmiR319* and *OsmiR396* are essential in BPH resistance networks [58,59,60,61,62]. Upon BPH attack, increased intracellular Ca^2+^ flux activates immune responses, promoting callose deposition in resistant varieties to strengthen cell walls and inhibit BPH infestation [63].

### 4.1. Phytohormones Signaling Functions in BPH Resistance

After a BPH attack, rice plants activate defense responses involving jasmonic acid (JA) and SA. Studies have shown that BPH feeding upregulates JA signaling pathway genes and significantly increases JA levels, while SA levels remain unchanged [64]. JA-deficient mutants, such as *AOC* and *MYC2* knockouts, exhibit reduced biosynthesis of secondary metabolites and increased susceptibility to BPH. In contrast, SA-deficient lines respond similarly to wild-type plants under BPH feeding, suggesting that JA plays a more critical role in this response [64]. In the BPH-resistant rice variety RH, SA pathway genes are upregulated after BPH feeding, with significant increases in SA levels and decreases in JA levels. This indicates that SA plays a leading role in this variety’s defense mechanism [65]. Suppressing *OsHI-LOX*, a lipoxygenase upregulated under BPH feeding, enhances resistance to BPH and is associated with elevated levels of H_2_O_2_ and SA, as well as increased cell death [66,67]. *Os6PGDH1* modulates JA, ethylene, and H_2_O_2_ levels in response to BPH feeding, affecting plant resistance [68]. The JA signaling transcription factor *Os*MYC2 enhances resistance by activating *OsCsLF6*, which regulates β-1,3;1,4-D-glucan synthesis, leading to the thickening of vascular bundle cell walls [69]. In rice, COI proteins *Os*COI1a, *Os*COI1b, and *Os*COI2 play crucial roles in mediating resistance to BPH [70]. Upon BPH attack, SA concentrations and transcription levels of resistance genes like *Bph14*, *Bph6*, *bph29*, and *Bph9* significantly increase, indicating an active response [38,43,45,47]. BPH feeding induces *OsHLH61* expression, which is promoted by MeJA and OPDA but inhibited by SA [71]. In *OsHLH61* RNAi plants, SA-mediated pathogenesis-related (PR) genes are downregulated, increasing susceptibility to BPH [71].

Ethylene plays a critical role in rice defense against BPH. BPH feeding rapidly activates the ethylene signaling pathway, leading to the upregulation of the *Brown Planthopper Induced008a* (*Bphi008a*) gene, while blocking ethylene signaling significantly reduces its transcription. Furthermore, the proline-rich region at the carboxyl terminus of Bphi008a interacts with rice Mitogen-activated Protein Kinase5 (*Os*MPK5) and undergoes phosphorylation in the nucleus [49]. Antisense expression of *OsACS2* reduces ethylene release and increases the emission of repellent volatiles, enhancing resistance to BPH [72]. *Os*EBF2 interacts with *Os*EIL1, regulating ethylene content and positively influencing resistance [73]. *OsERF3* responds to BPH feeding by modulating JA, SA, ethylene, and H_2_O_2_ pathways, affecting plant resistance [74]. Under dim light, decreased *OsPHYB* leads to increased ethylene production, lowering BPH resistance. Overexpression of *OsSAMS1* increases ethylene but diminishes resistance, indicating that ethylene negatively regulates defense against BPH [75].

In rice, the gibberellin (GA) receptor gene *OsGID1* is induced by BPH feeding, mechanical damage, and SA treatment, but not affected by JA. Studies have shown that overexpression of the *OsGID1* gene (*GID1*_OE) reduces the expression levels of SA, H_2_O_2_, and three SA pathway-related WRKY transcription factors induced by BPH feeding, while increasing ethylene levels and lignin content, thereby enhancing rice resistance to BPH [76]. DELLA proteins, negative regulators in the GA pathway, are present in rice. The rice DELLA gene *OsSLR1* positively regulates the expression of two mitogen-activated protein kinases and four WRKY transcription factors, as well as the levels of JA, ethylene, and H_2_O_2_, under BPH induction. Silencing of the *OsSLR1* gene increases the baseline levels of defense-related compounds, phenolic acids, lignin, and cellulose, thus enhancing rice’s resistance to BPH [77]. BPH feeding has been found to promote the biosynthesis and signaling of cytokinins (CKs) in rice. In rice plants treated exogenously with CK and where cytokinin oxidase/dehydrogenase (*OsCKXs*) genes were knocked out, the levels of JA and the expression of JA response genes were significantly upregulated, significantly enhancing resistance to BPH. Conversely, JA-deficient mutants, such as *og1*, exhibit increased susceptibility to BPH, and the CK-induced resistance against BPH is suppressed in these mutants, indicating that CK-mediated resistance is dependent on JA [78]. In rice, overexpression of *9-cis-Epoxycarotenoid Dioxygenase* (*NCED*), a rate-limiting enzyme in the abscisic acid (ABA) biosynthesis pathway, enhances resistance to BPH and increases the levels of ABA, JA, and JA-Ile induced by BPH [79]. Transcriptomic analysis reveals that differentially expressed genes are primarily associated with stress responses and JA biosynthesis [80]. Exogenous application of ABA or drought treatment leads to the accumulation of oligosaccharides in rice, which inhibits BPH feeding and oviposition, thereby enhancing resistance [81]. Furthermore, the nuclear-localized E3 ligase *OsJMJ715* is upregulated upon BPH feeding, negatively regulating the biosynthesis of ABA, JA, and JA-Ile, as well as the deposition of callose, while BPH promotes the expression of *OsJMJ715* to obtain more nutrients [82].

### 4.2. Metabolites

Upon BPH infestation, rice plants alter primary metabolites—such as sugars, organic acids, amino acids, and choline—especially in susceptible varieties. In resistant varieties, key genes regulating the GABA shunt and secondary metabolite synthesis are upregulated, highlighting the role of the GABA shunt and oxalate-mediated metabolism in resistance [83]. Genes involved in the phenylpropanoid pathway, including *Os*C4H, *Os*CHS, and *Os*CHI, are activated, enhancing defense responses [51]. Phenylalanine ammonia-lyase (PAL), upregulated by the R2R3 MYB transcription factor *Os*MYB30, increases SA and lignin synthesis, strengthening resistance [84]. Additionally, overexpression of *OsGRF8*, a target of *OsmiR396*, induces flavonoid 3-hydroxylase (*Os*F3H) and flavonoid accumulation, enhancing BPH resistance through the *Os*miR396-*Os*GRF8-*Os*F3H–flavonoid pathway [60]. JA signaling, mediated by *MYC2*, promotes sakuranetin synthesis via *OsNOMT*, further combating BPH invasion [85].

In response to BPH infestation in rice, it is particularly noteworthy that the BPH30T rice variety exhibits a general downregulation of amino acids and their derivative metabolic products (DAMs), while most flavonoid metabolites show an increasing trend. In contrast, an opposite pattern is observed in the Nipponbare rice variety, suggesting that the *Bph30* gene may regulate the plant’s primary and secondary metabolites and hormone transport through the oxalate pathway, thereby enhancing resistance to BPH [86]. Additionally, jasmonates (JAs) and green leaf volatiles (GLVs) serve as important signaling molecules in plant defense against pests and diseases, playing multiple functional roles. In rice, the hydroperoxide lyase (HPL) *Os*HPL3/CYP74B2 can hydrolyze hydroperoxy linolenic acid to produce GLVs. Studies have found that during this process, the levels of (Z)-3-hexen-1-ol are increased, while the induction of JA, protease inhibitors, and other volatiles is diminished, ultimately positively regulating rice resistance to BPH [87]. Inducible S-linalool can attract carnivorous animals, parasitic wasps, and chewing herbivores while repelling BPH; conversely, the constitutive (E)-β-caryophyllene attracts parasitic wasps and planthoppers but repels other herbivores [88].

In the chemical defense mechanisms of rice, the enzyme serotonyl 5-hydroxylase, encoded by the cytochrome P450 gene *CYP71A1*, is pivotal, converting serotonin into 5-hydroxyserotonin. In susceptible wild-type rice, BPH feeding induces the biosynthesis of 5-hydroxyserotonin and SA. However, in mutants lacking the *CYP71A1* gene, the production of 5-hydroxyserotonin is blocked, and elevated levels of SA enhance the plants’ insect resistance. Notably, the addition of 5-hydroxyserotonin to resistant rice mutants and other rice varieties with BPH resistance traits reduces their insect resistance [89]. Following BPH feeding, rice also induces the production of phenylamides (PAs), particularly cadaverine derivatives such as coumaroyl cadaverine and feruloyl cadaverine, to defend against the pest [90]. Furthermore, overexpression of the xylanase inhibitor *OsHI-XIP* reduces BPH feeding and oviposition preferences without affecting rice plant growth and development [91]. *Os*RIP1, a ribosome-inactivating protein, is significantly upregulated during BPH feeding, with transcription levels in infested plants being 100 times higher than in uninfested plants. Although recombinant *Os*RIP1 is toxic to BPH, its concentration in the phloem of rice may not be sufficient to significantly impact feeding [92]. In plant resistance mechanisms, the amino acid transporter *Os*ATL15 is located in the rice cell membrane and is highly expressed in root cross-sections, leaf vascular bundles, and stem longitudinal sections. It facilitates the systemic distribution of thiamethoxam in rice and effectively inhibits BPH feeding [93]. Additionally, *Os*LCB2a1, encoding a subunit of serine palmitoyl transferase, a key enzyme in sphingolipid biosynthesis, is upregulated during BPH feeding, enhancing the plant’s defense against herbivores [94]. The rice peptide *Os*Pep3 is induced by BPH feeding, and the exogenous application of *Os*Pep3 regulates JA biosynthesis, lipid metabolism, and phenylpropanoid metabolism, thereby increasing rice resistance to pests [95].

### 4.3. Functions of Abiotic Factors in BPH Resistance

In rice, the defense against BPH feeding involves complex gene regulation, with several key genes playing crucial roles. SiO_2_ engineered nanomaterials (ENMs) can induce rice to enhance lignin synthesis, strengthen cell walls, form silica cells, and increase the synthesis of long-chain waxes, thereby improving resistance to BPH. At a concentration of 5 mg L^−1^ of F-SiO_2_ ENMs, an upregulation of rice genes associated with pest resistance is also observed. SiO_2_ ENMs, as a novel silica-based nanopesticide, hold broad prospects for development in sustainable agriculture [96]. For genetically modified *Bacillus thuringiensis* (*Bt*) rice, studies show that after continuously feeding on transgenic rice expressing the Cry1Ab protein for two generations, the long-winged subpopulation of BPH increases with elevated CO_2_ concentrations, while the short-winged subpopulation significantly decreases. At ambient temperature, *Bt* rice significantly inhibits the activity of peroxidase (POD) and superoxide dismutase (SOD); however, at high CO_2_ concentrations, only the activity of SOD is significantly enhanced. Additionally, high CO_2_ concentrations significantly reduce the number of YLES in female short-winged BPH, but the effect on male short-winged BPH feeding on transgenic rice is only a significant reduction [97].

Research indicates that following BPH feeding, the *SWEET13/14* genes mediate the transport of sugars from the leaves to the phloem sap, facilitating BPH feeding. Mutants of *sweet13/14* not only show enhanced resistance to bacterial blight and BPH but also do not negatively impact yield [98]. Additionally, after BPH feeding, the expression levels of the *OsBi1* gene in rice increase, a response associated with ethylene and water deficit. In situ hybridization studies show that *OsBi1* transcripts specifically accumulate around the vascular bundles in the stem, and this gene’s expression is closely related to rice resistance to BPH [99]. The *OsLOX1* gene has low expression in immature seeds and just-germinated seedlings of rice but rapidly accumulates in response to mechanical damage or BPH feeding, reaching peak expression at 3 h after injury and 6 h after BPH feeding. When *OsLOX1* expression is low, rice’s resistance to BPH is diminished. Conversely, enhanced expression of *OsLOX1* strengthens its resistance [100]. The *OsTPS31* promoter (Ptps31) and its 7 bp cis-regulatory sequence are unresponsive to physical damage but are activated by BPH feeding. These elements facilitate the upregulation of downstream genes following BPH feeding, offering new tools for the study and application of BPH resistance-related genes [101]. Additionally, the epidermis-specific protein ACL1 interacts with ROC1-8 and rice TOPLESS-related proteins (TPRs), suggesting that ACL1 may recruit TPRs to repress the transcription of ROC4/5, thereby regulating rice cuticle wax content and bulliform cell development, enhancing drought and BPH resistance [102]. Furthermore, it was discovered that the leucine-rich repeat protein *Os*LRR2 interacts with the co-receptors *Os*SERK1 and *Os*SERK2, located on the cell membrane. This interaction disrupts the formation of complexes between defense-related pattern recognition receptors (e.g., *Os*PEPR1 and *Os*FLS2) and growth-related receptors (e.g., OsBRI1), thus inhibiting both defense responses and growth in rice. Knockout of *OsLRR2* significantly enhanced rice resistance to BPH and blast disease and increased the response to bacterial elicitor flg22, while also boosting rice yield [103]. Additionally, knockout of the leucine-rich repeat receptor-like kinase *Os*LRR-RLK18 led to reduced plant height and root length, and later stages of BPH infestation triggered significant increases in JA, JA-Ile, and ABA levels, while SA and hydrogen peroxide levels decreased during early infestation. This response was accompanied by increased volatile release, reduced lignin and flavonoid content, and significantly lower BPH egg hatching rates and oviposition, indicating that *Os*LRR-RLK18 plays a critical role in rice defense against BPH.

**Table 1:** Rice BPH resistance-related genes.

Pathway	Genes	Accession Numbers	Gene Annotation	References
MAPK signaling pathway	*OsMPK1*	LOC_Os06g06090	Mitogen-activated protein kinase	[51]
	*OsMPK3*	LOC_Os02g05480	Mitogen-activated protein kinase	[50,51]
	*OsMPK4*	LOC_Os06g48590	Mitogen-activated protein kinase	[51]
	*OsMPK5*	LOC_Os03g17700	Mitogen-activated protein kinase	[49,51]
	*OsMPK7*	LOC_Os05g49140	Mitogen-activated protein kinase	[51]
	*OsMPK8*	LOC_Os01g47530	Mitogen-activated protein kinase	[51]
	*OsMPK9*	LOC_Os05g50560	Mitogen-activated protein kinase	[51]
	*OsMPK12*	LOC_Os06g49430	Mitogen-activated protein kinase	[49,51]
	*OsMPK13*	LOC_Os02g04230	Mitogen-activated protein kinase	[49,51]
	*OsMPK14*	LOC_Os02g05480	Mitogen-activated protein kinase	[51]
	*OsMPK16*	LOC_Os01g45620	Mitogen-activated protein kinase	[51]
	*OsMPK17*	LOC_Os05g50120	Mitogen-activated protein kinase	[49]
	*OsMKK3*	LOC_Os06g27890	Mitogen-activated protein kinase kinase	[52]
Transcription factor	*OsbHLH065*	LOC_Os04g41570	Phosphorylated substrate of OsMPK3	[50]
	*OsbZIP60*	LOC_Os07g44950	bZIP transcription factor	[49]
	*OsSPL10*	LOC_Os06g44860	Squamosa promoter binding protein	[57]
	*OsMYB30; OsMYB5P*	LOC_Os02g41510	MYB transcription factor	[84]
	*OsWRKY45*	LOC_Os05g25770	WRKY transcription factor	[55]
	*OsWRKY53*	LOC_Os05g27730	WRKY transcription factor	[54]
	*OsMYB22*	LOC_Os01g65370	MYB transcription factor	[58]
	*OsI-BAK1*	LOC_Os08g07760	SERK family of receptor-like protein kinases	[56]
	*OsWRKY70*	LOC_Os05g39720	WRKY transcription factor	[56]
miRNA	*OsmiR319*	-	BPH resistance-related gene	[58]
	*OsmiR396*	-	BPH resistance-related gene	[60]
	*OsGRF8*	LOC_Os11g35030	Growth regulatory factor	[60]
	*OsF3H*	LOC_Os03g03034	Flavanone 3 beta-hydroxylase	[60]
	*OsmiR156*	-	BPH resistance-related gene	[59]
	*OsmiR159*	-	BPH resistance-related gene	[62]
	*Osa-miR162a*	-	BPH resistance-related gene	[61]
Phytohormones	*OsAOC*	LOC_Os03g32314	Allene oxide cyclase	[64]
	*OsMYC2*	LOC_Os10g42430	Basic helix–loop–helix transcription factors	[64]
	*OsNOMT*	LOC_Os12g13800	Naringenin 7-O-methyltransferase	[85]
	*OsAOS2; AOS*	LOC_Os03g12500	Allylene oxide synthase	[104]
	*OsAOS1*	LOC_Os03g55800	Allylene oxide synthase	[67]
	*OsHI-LOX; OsLOX9*	LOC_Os08g39840	Lipoxygenase gene	[66]
	*OsJMT1*	LOC_Os06g20920	Jasmonate carboxymethyl transferase	[105]
	*Os6PGDH1*	LOC_Os06g02144	6-phosphogluconate dehydrogenase	[68]
	*OsCsLF6*	LOC_Os08g06380	Cellulose-like synthase F	[69]
	*OsCOI1; OsCOI1a*	LOC_Os01g63420	F-box protein, jasmonic acid receptor	[106,107]
	*OsCOI1b*	LOC_Os05g37690	Principal component of a receptor of JA	[70]
	*OsCOI2*	LOC_Os03g15880	Jasmonate receptor	[70]
	*OsPAL6*	LOC_Os04g43800	Phenylalanine ammonia-lyase	[84]
	*OsPAL8*	LOC_Os11g48110	Phenylalanine ammonia-lyase	[84]
	*OsNPR1*	LOC_Os01g09800	SA receptor	[108]
	*OsNPR3*	LOC_Os03g46440	SA receptor	[108]
	*OsNPR4*	LOC_Os01g61990	SA receptor	[108]
	*OsHLH61*	LOC_Os07g47960	Helix–loop–helix protein	[71]
	*OsbHLH96*	LOC_Os03g08930	bHLH transcription factor	[71]
	*Bphi008a*	LOC_Os06g29730	BPH resistance gene	[49]
	*OsOPR7; OPR3*	LOC_Os08g35740	12-oxygen-plant dienolate reductase	[72]
	*OsACS2*	LOC_Os04g48850	1-aminocyclopropane-1-carboxylate synthase	[72]
	*OsEBF2*	LOC_Os02g10700	OsFBL7-F-box domain and LRR containing protein	[73]
	*AP37; OsERF3*	LOC_Os01g58420	Ethylene response factor	[74]
	*OsEIL2*	LOC_Os07g48630	Ethylene signaling regulatory factors	[109]
	*PHYB; OsphyB*	LOC_Os03g19590	Photochrome B	[75]
	*OsEBF1*	LOC_Os06g40360	E3 ubiquitin ligase	[75]
	*OsSAMS1*	LOC_Os05g04510	S-adenosine-l-methionine synthase 1	[75]
	*GID1; OsGID1*	LOC_Os05g33730	GA is not sensitive to dwarf genes	[76]
	*OsSLR1*	LOC_Os03g49990	Slender stalk gene	[77]
	*OsCKX1*	LOC_Os01g09260	Cytokinin oxidase/dehydrogenase	[78]
	*OsNCED3*	LOC_Os03g44380	9-*cis*-epoxide carotenoid dioxygenase	[78]
	*OsJMJ715*	LOC_Os03g31594	RING E3 ubiquitin ligase	[82]
Metabolites	*OsC4H*	LOC_Os05g25640	Cytochrome P450	[51]
	*OsCHS*	LOC_Os11g32650	Chalcone synthase	[51]
	*OsCHI*	LOC_Os03g60509	Chitinase	[51]
	*OsHPL3*	LOC_Os02g02000	Lip hydroperoxide lyase	[87]
	*CYP71A1*	LOC_Os12g16720	Tryptamine hydroxylase	[89]
	*OsHI-XIP*	LOC_Os05g15770	Xylanase inhibitor gene	[91]
	*OsRIP1; OsjRIP1.1*	LOC_Os11g06460	Ribosome inactivating protein	[92]
	*OsATL15*	LOC_Os01g41420	Transmembrane amino acid transporter protein	[93]
	*OsLCB2a1*	LOC_Os01g70380	Serine palmitoyl transferase 2	[94]
	*OsPep3*	LOC_Os08g07660	Plant elicitor peptide	[95]
Biotic and abiotic factors	*OsPIL14*	LOC_Os07g05010	Photochrome interaction factor	[75]
	*OsACO1*	LOC_Os03g04410	1-aminocyclopropane-1-carboxylate oxidase	[75]
	*SWEET13*	LOC_Os12g29220	Sucrose transporter	[98]
	*SWEET14*	LOC_Os11g31190	Sucrose transporter	[98]
	*OsBi1*	LOC_Os02g57280	CBS-like domain, Cystathione-beta synthase	[99]
	*OsLOX1*	LOC_Os03g49380	Lipoxygenase gene	[100]
	*TPS46; OsTPS31*	LOC_Os08g07100	Terpene synthase	[101]
	*OsSAMSL*	LOC_Os07g29440	S-adenosine-l-methionine synthetase 2	[46]
	*OsExo70E1*	LOC_Os01g55799	Exo70 exocyst complex subunit domain containing protein	[45]
	*OsExo70H3*	LOC_Os12g01040	Exo70 exocyst complex subunit domain containing protein	[46]
	*ACL1*	LOC_Os04g33860	Abaxially curled leaf 1	[102]
	*ROC4*	LOC_Os04g48070	Homeodomain leucine zipper IV transcription factor	[102]
	*ROC5*	LOC_Os02g45250	Homeodomain leucine zipper class IV gene	[102]
	*OsLRR2*	LOC_Os11g31540	Leucine-rich repeat protein	[103]
	*OsSERK1*	LOC_Os08g07760	SERK-family receptor-like protein kinase	[103]
	*OsSERK2*	LOC_Os04g38480	SERK-family receptor-like protein kinase	[103]
	*OsPEPR1*	LOC_Os08g34640	Plant elicitor peptide receptor	[95]
	*OsFLS2*	LOC_Os04g52780	Leucine-rich repeat receptor protein kinase EXS precursor	[103]
	*OsBRI1*	LOC_Os01g52050	BR receptor kinase	[103]

## 5. Complex Interaction Mechanism Between BPH Saliva Components and Rice Defense Response

Plants and herbivorous insects have developed intricate defense and counter-defense strategies through long-term coevolution. Rice plants recognize Herbivore-Associated Molecular Patterns (HAMPs), derived from insect secretions, eggs, and surface secretions, to trigger immune responses. These include fatty acid–amino acid conjugates (e.g., volicitin), oxylipins, peptides, and enzymes [110]. Insects, in turn, secrete effectors to suppress plant defenses, escalating the evolutionary arms race. Piercing–sucking insects like BPH produce two types of saliva from their salivary glands: gel saliva, which forms a hardened sheath for stable feeding, and watery saliva, rich in digestive enzymes such as pectinase and cellulase, which is reabsorbed with plant sap. Insect saliva also contains elicitors and effectors that respectively activate or suppress plant defenses, shaping the coevolution between rice and BPH. In rice–BPH interactions, most resistance genes encode proteins like CC-NB-LRR and LecRK that recognize BPH salivary effectors. For example, BISP, a BPH effector, suppresses rice defenses by binding to *Os*RLCK185. However, in rice with the *Bph14* gene, BISP is recognized and degraded through autophagy, triggering strong defenses while maintaining rice yield [42]. Some salivary proteins can simultaneously promote and suppress plant defenses. Professor Jianping Chen’s team discovered LsMLP, a major component of the salivary sheath of the white-backed planthopper (WBPH), which lubricates and protects the stylet but also activates rice defenses. To evade this, WBPH secretes LsSP1, which surrounds LsMLP to avoid immune recognition and disrupts the cysteine protease and SA pathway, further suppressing rice defenses [111]. Understanding the role of BPH salivary proteins not only elucidates the coevolutionary dynamics between rice and BPH but also provides insight into the formation of BPH biotypes and their pathogenicity.

### 5.1. Interaction Between BPH Saliva and Rice

With the rapid advancement of omics technologies, researchers have adopted various approaches to study the salivary proteins of BPH. Transcriptome sequencing of salivary glands from BPH populations with different virulence levels predicted 352 genes potentially encoding secreted proteins [112]. By combining artificial feeding systems with liquid chromatography–tandem mass spectrometry (LC-MS/MS), proteomic data for gel-like and water-soluble saliva were obtained, identifying 107 major water-soluble salivary proteins. Among these, 29 exhibited catalytic activity, while 24 were binding proteins, including oxidoreductases, hydrolases, phosphatases, peptidases, kinases, transferases, and lyases [113]. Furthermore, RNA interference experiments identified key protein factors critical for the formation of salivary sheaths [114].

**Figure 2:**
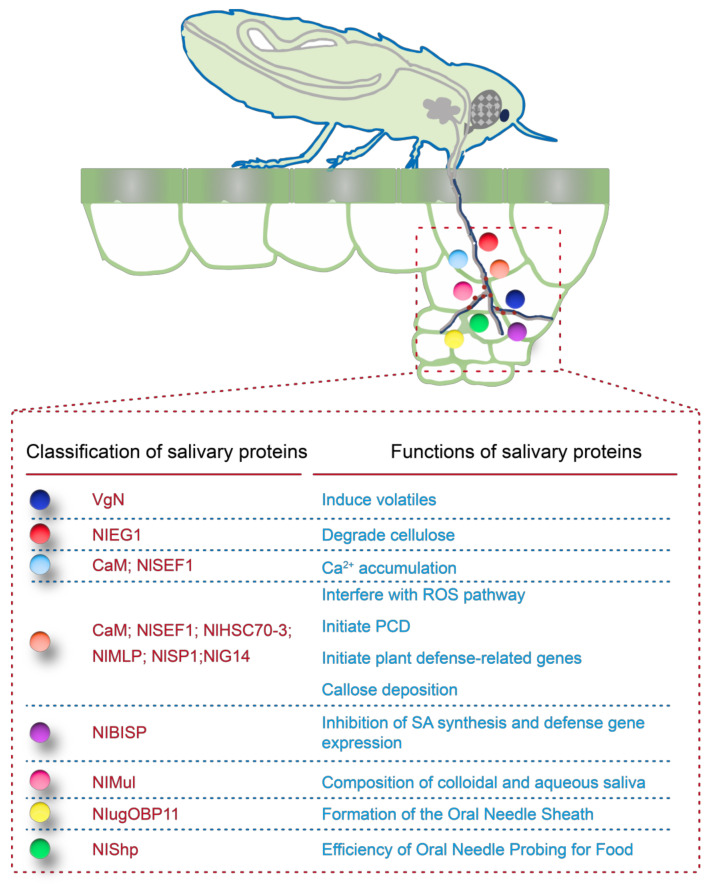
Functional characterization of salivary proteins secreted by BPH while feeding on rice plants. This figure illustrates the role of various salivary proteins secreted by BPH, which are critical in facilitating feeding and manipulating host plant responses. The top panel shows a schematic of a BPH feeding on a rice plant, highlighting the injection of salivary proteins into plant tissue. The bottom panel provides a classification and functional annotation of these proteins, detailing their diverse roles in inducing plant volatiles, degrading cellulose, interfering with calcium and ROS pathways, initiating plant cell death, and manipulating plant defense mechanisms. Each protein’s function is marked by a unique color, aligning with the key provided, which helps in visually distinguishing their specific roles in BPH–plant interactions.

In the long-term coevolution between plants and herbivorous insects, complex interactions have developed, exemplified by the relationship between rice and BPH. Rice recognizes elicitors in BPH saliva, triggering early signaling events such as increased cytoplasmic calcium levels, reactive oxygen species (ROS) bursts, and activation of the MAPK cascade. These events activate defense-related hormonal pathways—including JA, SA, ethylene, abscisic acid, and gibberellins—and promote the production of primary metabolites like amino acids and secondary metabolites such as oxalic acid, oryzalexins, protease inhibitors, and flavonoids, thereby enhancing rice resistance to insects (Figure 2) [105].

Several elicitors from BPH saliva have been identified. Mucin-like proteins (MLPs) are key components of the salivary sheath. These proteins are highly homologous, and their knockdown significantly affects the formation and survival rate of the salivary sheath [115]. Another elicitor, the N-terminal peptide of vitellogenin (VgN), triggers strong defense responses when introduced into the plant during feeding or oviposition, reducing the BPH hatching rate and promoting the release of volatile metabolites that attract egg parasitoids.

BPH head extracts containing β-glucosidase induce rice to produce volatiles that attract parasitic wasps, enhancing natural control of the pest [116]. Endo-β-1,4-glucanase in BPH saliva degrades the cellulose in rice cell walls, facilitating nutrient access from the phloem [117]. During feeding, BPH releases hydrogen peroxide-like proteins, calmodulin-binding protein NISEF1, and calmodulin CaM, which neutralize plant-produced hydrogen peroxide and disrupt ROS defense responses, evading callose deposition and improving feeding success [118,119,120]. The salivary sheath protein NIShp is critical for sheath formation; silencing its gene increases probing frequency and reduces sap intake [121].

Odor-binding protein NlugOBP11 transports odor molecules, aiding BPH colonization and possibly inhibiting SA production in plant defenses. Other salivary proteins—such as heat shock protein NIHSC70-3, mucin-like proteins NIMul and NIMLP, salivary protein NISP1, and saliva-specific protein NIG14—play roles in inducing plant cell apoptosis, stimulating defense gene expression, and callose deposition, potentially activating the JA signaling pathway. The DNAJ protein NlDNAJB9, highly expressed in salivary glands, affects honeydew excretion and fecundity and induces plant cell death. Overexpression of NlDNAJB9 in Nicotiana benthamiana activates calcium signaling, MAPK cascades, ROS accumulation, JA signaling, and callose deposition, inhibiting insect feeding and pathogen infection. NlDNAJB9 likely regulates plant defense by interacting with NlHSC70-3, inducing ROS bursts and cell death. Odor-binding protein NlugOBP11 transports odor molecules, aiding BPH colonization and possibly inhibiting SA production in plant defenses [122]. Other salivary proteins—such as heat shock protein NIHSC70-3, mucin-like proteins NIMul and NIMLP, salivary protein NISP1, and saliva-specific protein NIG14—play roles in inducing plant cell apoptosis, stimulating defense gene expression, and callose deposition, potentially activating the JA signaling pathway [115,123,124,125,126]. The DNAJ protein NlDNAJB9, highly expressed in salivary glands, affects honeydew excretion and fecundity and induces plant cell death. Overexpression of NlDNAJB9 in Nicotiana benthamiana activates calcium signaling, MAPK cascades, ROS accumulation, JA signaling, and callose deposition, inhibiting insect feeding and pathogen infection. NlDNAJB9 likely regulates plant defenses by interacting with NlHSC70-3, inducing ROS bursts and cell death [127]. Gel-like salivary proteins NlSP5 and NlSP7 are specific to BPH. Functional knockdown of these proteins reduces BPH adaptability, leading to prolonged development, reduced lifespan, lower body weight, fertility, and hatching rates. Flonicamid inhibits BPH feeding by suppressing salivary protein genes such as *NlShp*, *NlAnnix5*, *Nl16*, *Nl32*, and *NlSP7* [128].

Recent studies have revealed that BPH saliva contains microRNAs (miRNAs) capable of crossing species barriers to regulate plant defense responses. Five salivary miRNAs—miR-100-5p, miR-7-5p, miR-184-3p, miR-1-3p, and miR-9a-5p—are abundantly secreted into plant tissues. Silencing miR-7-5p in BPH induces the expression of the defense transcription factor bZIP43 in rice, triggering insect resistance. This finding offers new insights into plant–insect coevolution and interaction mechanisms [129]. Traditionally considered exocrine, insect salivary glands have been found to possess endocrine functions in some species, such as fruit flies [130]. In BPH, the salivary protein NlG14 affects the endocrine function of salivary glands. Reduced levels of NlG14 disrupt insulin-like peptide (NlILP1 and NlILP3) synthesis and secretion in the brain, activating the insulin–PI3K-Akt and ecdysone signaling pathways in the ovaries. This leads to the mislocalization of LOSC in ovaries, ultimately inhibiting ovulation. This discovery highlights the endocrine role of salivary glands in BPH and their impact on reproduction [131]. Overall, saliva plays a crucial role in the coevolution between BPH and rice, influencing plant defense mechanisms and insect adaptability. Understanding these interactions at the molecular level provides valuable insights for developing new strategies in pest management and enhancing crop resistance.

### 5.2. Other BPH Effectors

During the interaction between rice and BPH, the eggs, egg-associated secretions (EAS), and honeydew elicit defense responses in rice. During oviposition, EAS deposited in damaged rice tissues triggers the accumulation of JA, JA-isoleucine, and hydrogen peroxide, enhancing rice defenses against BPH [132]. Vitellogenin (Vg), essential for insect embryo development, plays a pivotal role in this process. Specifically, the N-terminal subunit of BPH vitellogenin (NlVgN), present in salivary secretions and on egg surfaces, enters rice tissues during feeding and oviposition. NlVgN acts as an elicitor by inducing rises in cytosolic calcium ions and hydrogen peroxide levels and stimulating the biosynthesis of JA and JA-Ile [133]. The defense responses induced by NlVgN not only decrease BPH hatching rates but also cause rice to emit volatiles that attract egg parasitoids, enhancing biological control. Rice responses to NlVgN differ between feeding and oviposition, suggesting that rice tailors its defenses to the insect’s developmental stages. This highlights potential evolutionary adaptations by BPH to evade detection by rice plants [133].

## 6. BPH-Mediated Rice Virus Transmission and RNA Interference-Based Resistance Strategies

BPH not only directly harms rice plants but also transmits two major viruses: Rice grassy stunt virus (RGSV) and Rice ragged stunt virus (RRSV). RGSV is a negative-sense single-stranded RNA virus, while RRSV is a double-stranded RNA virus. Both viruses are transmitted in a persistent–propagative manner by BPH, leading to severe symptoms in rice, including stunted growth, excessive tillering, and failure to produce panicles, which significantly reduce rice yield. BPH carrying RRSV exhibits several physiological changes: shortened nymphal stages, increased proportions of females and short-winged adults, extended adult lifespans, and significantly enhanced reproductive capacity. The expression of genes related to reproduction, such as *Vg* and *VgR*, is significantly upregulated, boosting the insect’s fecundity and virus transmission capacity [134].

Studies on rice antiviral mechanisms have shown that RNA interference (RNAi)-mediated antiviral pathways play critical roles in plant–virus interactions. Silencing BPH genes *NlKPI* or *NlVenomase* using dsRNA significantly increases the mortality of *N. lugens* infected with Metarhizium fungus. These genes regulate the expression of antimicrobial peptides and phenoloxidase activity, contributing to the insect’s immune response to both RRSV and fungal infection [135]. Although RNA interference (RNAi)-based antiviral strategies have successfully developed rice germplasm resistant to RRSV and RGSV [136], evidence linking these strategies to direct BPH resistance remains insufficient. Future research should further explore the relationship between antiviral and anti-BPH mechanisms, providing insights for more integrated pest management strategies in rice production.

## 7. Development and Improvement of BPH-Resistant Rice Varieties

BPH has demonstrated a rapid adaptation to rice varieties, particularly in overcoming resistance genes. The International Rice Research Institute (IRRI) released the BPH-resistant rice varieties IR26 and IR36 in 1973 and 1976, respectively. However, the resistance of these varieties declined over a few years of cultivation due to the evolution of BPH biotypes. In response, IRRI developed several new varieties carrying resistance genes *Bph1*, *Bph2*, *Bph3*, and *Bph4*, from IR28 to IR74, totaling 16 varieties [33]. In the 1970s, China also began to emphasize the identification and breeding of BPH-resistant rice resources. Through in-depth studies of hybrid vigor in rice resistance to BPH, Chinese researchers utilized a cross between “Zhenshan 97A” and the IR26 restorer line to successfully develop China’s first BPH-resistant indica hybrid rice, effectively reducing BPH damage in rice-producing areas. Subsequently, a series of BPH-resistant hybrid rice varieties such as “Shanyou 30”, “Shanyou 54”, “Nanyou 6”, and “Liuyou 30” were promoted. Although these varieties mostly carry the *Bph1* and *Bph2* resistance genes, there is a risk of BPH gradually adapting to these genes, thus diminishing the effectiveness of the rice’s insect resistance.

Due to the limited availability of BPH-resistant rice resources, Professor He Guangcun’s research team at Wuhan University has made significant achievements in cloning BPH resistance genes. They successfully cloned *Bph14*, the world’s first rice BPH resistance gene, and, to date, have cloned 60% of the world’s known BPH resistance genes. These genes are not only resistant to various BPH biotypes but also show high resistance to the white-backed planthopper, especially effective in rice varieties carrying *Bph14*, *Bph15*, *Bph6*, or *Bph30*. Notably, *Bph6* and *Bph30* are fully dominant genes. In rice hybrid breeding, even heterozygous offspring demonstrate the same level of insect resistance as homozygous individuals, making these genes particularly valuable in breeding applications. Utilizing these resistance genes, the research team has developed a series of new BPH-resistant rice varieties. These varieties have been extensively adopted across multiple provinces including Hubei, Anhui, Hunan, Jiangxi, Guangxi, and Hainan. The results show that the promotion of these resistant rice varieties has significantly reduced the field density of BPH, with a reduction rate exceeding 85% [137]. *Bph27*, *Bph35*, and *Bph42* have all been widely applied in rice breeding through molecular marker-assisted selection (MAS) technology. These genes are located on the fourth chromosome of rice. *Bph27*, derived from wild rice, reduces BPH feeding and reproduction through antibiosis and antixenosis resistance mechanisms [138]. *Bph35*, sourced from the rice genetic resource pool, enhances rice’s immune response by activating defense signaling pathways such as MAPK, jasmonic acid, and gibberellin, thereby suppressing BPH growth and reproduction [139]. *Bph42*, a newly discovered resistance gene, regulates immune responses and hormone signaling to mitigate BPH infestation and inhibit its growth and reproduction [140]. To improve resistance durability, *Bph27* is often used in combination with *Bph35* and *Bph42* for gene pyramiding, and these combinations have been introduced into multiple commercial rice varieties. This multi-gene approach enhances resistance to different BPH biotypes and slows the breakdown of resistance.

The application of single-gene resistance in rice faces challenges due to the adaptive evolution of BPH, leading to the loss of resistance in rice. To overcome this challenge, Chinese breeders have combined molecular marker-assisted selection with conventional backcross breeding to effectively shorten the generational intervals and achieve early selection, thereby accelerating the breeding process for BPH resistance genes. This method has successfully introduced multi-gene resistance, including single-, double-, triple-, and quadruple-gene resistance into rice, resulting in a series of improved BPH-resistant rice varieties. Wuhan University utilized the elite two-line sterile line “Guangzhan 63-4S” as the female parent, and through crossing with high BPH-resistant materials carrying *Bph14* and *Bph15*, followed by multiple generations of backcrossing and combining molecular marker-assisted selection, successfully developed the new two-line sterile line Bph68S with strong combinatorial ability and excellent BPH resistance. Molecular marker-assisted selection techniques were successfully used to introduce *Bph3* and *Bph14* resistance genes into the Gui Nong Zhan variety, obtaining several stable lines containing these resistance genes. Additionally, by introducing *Bph14* and *Bph15* into the sensitive restorer line Hua Hui 938, the BPH resistance of this restorer line and its derived hybrid rice varieties was significantly enhanced, with offspring screened using the RICE6K chip for genetic traits [141]. At Huazhong Agricultural University, the team led by Professor Dabing Yang employed genomic breeding techniques to aggregate four resistance genes, *Pi2*, *Xa23*, *Bph14*, and *Bph15*, from three photoperiod-temperature-sensitive male sterile donors into the Feng 39S variety. This photoperiod-temperature-sensitive male sterile variant has significantly enhanced comprehensive resistance to blast disease, bacterial blight, and BPH while maintaining excellent agronomic traits and grain quality [142]. Furthermore, *Bph9*, *Bph6*, *Bph15*, and *Bph14* genes were individually and collectively introduced into the drought-resistant restorer line Hanhui 3, with *Bph9* showing the strongest resistance. In the improved series, resistance was superior in the multi-gene modified lines, with the four-gene aggregated line exhibiting the most significant resistance. Under natural conditions, the improved varieties showed no significant differences compared to Hanhui 3 in plant height, effective panicles, and grain weight [143]. Finally, using multi-parent composite crossing techniques, donor parents harboring *Bph3* or *Bph24(t)* were crossed with high-quality three-line hybrid rice maintainer and restorer lines carrying blast disease resistance genes (*Pi2*, *Pib*, or *Pimh*), bacterial blight resistance genes (*Xa23*), or the fragrance gene (*badh2*), successfully aggregating BPH resistance genes into high-quality rice lines [144].

Recently, China has successfully developed and approved a batch of rice varieties with high resistance to BPH, which are now widely used in agricultural production. Wuhan University’s “Yiliangyou 311” and Huazhong Agricultural University’s “Hualiangyou 2171” carry the *Bph14* and *Bph15* genes. “Weiliangyou 7713” and “Luoyang 69” are rice lines carrying both *Bph6* and *Bph9* resistance genes, exhibiting high resistance to BPH. Among them, “Weiliangyou 7713” has been approved for cultivation in multiple regions, including the Yangtze River Basin and parts of southern China. By 2023, its cultivation area had expanded to over 53,300 hectares, making it an important resource for rice breeding programs focused on developing BPH-resistant varieties [25,145]. On the other hand, “Luoyang 69” is widely used as a resistant control in breeding programs to evaluate the BPH resistance of new rice varieties. Incorporating “Luoyang 69” into breeding projects has not only accelerated the development of BPH-resistant rice varieties but also ensured the stability of high yield and superior grain quality in these new lines [146]. Although multiple BPH resistance genes have been successfully identified and cloned, technically aggregating these genes in an effective and efficient manner remains challenging. This is why relatively few rice varieties in China have resistance to two or more pests and diseases. According to the latest 2023 national rice variety approval data released by China’s Ministry of Agriculture and Rural Affairs (see Announcement No. 726), out of 409 national rice varieties, only one variety exhibits high resistance to BPH, and two varieties show a moderate level of resistance. Additionally, only two varieties exhibit resistance to two different pests and diseases (such as blast, bacterial blight, and BPH) at a moderate or higher level. Among them, “Keguiyou 4302” is the first approved high-quality rice variety in China with resistance to blast, bacterial blight, and BPH, showing a yield increase of 1.9% compared to the control. The new hybrid mid-season rice variety “Chengliangyou 4312,” bred by China National Seed Group Co., Ltd., shows moderate resistance to blast and BPH, with a yield increase of 4.1% compared to the control variety. Furthermore, “Bliangyou 164” (carrying *Bph14* and *Bph15*), bred by the Sanming Agricultural Science Institute in 2019, is a stable, high-yielding, moderately blast- and BPH-resistant premium rice variety. Its grain quality meets the national standards for high-quality rice (Grade III according to national standards), suitable for planting as mid-season rice in the middle and lower reaches of the Yangtze River and as late rice throughout Fujian province [147].

## 8. Summary and Outlook

### 8.1. Research on Rice and BPH Resistance and Breeding Challenges

The long-term coevolution between BPH and rice has led to the emergence of various BPH biotypes, often resulting in the loss of resistance in previously resistant rice varieties [27]. To address this issue, researchers have identified 80 BPH resistance genes or QTLs in both cultivated and wild rice. Notably, Professor He Guangcun’s team at Wuhan University has been instrumental in cloning the world’s first BPH resistance gene, *Bph14*, accounting for 60% of globally cloned BPH resistance genes [38]. Advancements in rice genomics have enabled the cloning of 17 BPH resistance genes using map-based techniques, primarily encoding CC-NB-LRR or CC-NB-NB-LRR proteins. Practices at the International Rice Research Institute (IRRI) demonstrate that cultivating rice varieties with different resistance genes can effectively control BPH populations and slow the evolution of BPH biotypes, achieving durable resistance.

Rice pest management faces significant challenges. First, most BPH resistance genes confer only low levels of resistance, limiting their effectiveness. Second, resistance genes from diverse geographical and genetic backgrounds result in variable performance due to environmental and genetic factors, complicating breeding efforts. Third, traditional breeding methods rely on time-consuming hybridization and backcrossing, and introducing resistance genes without affecting other agronomic traits is critical. Fourth, dominance relationships affect resistance levels; heterozygous offspring often exhibit significantly weaker resistance compared to homozygous types. Fifth, while research on BPH resistance mechanisms is extensive, there is a lack of in-depth exploration of the interactions between resistance genes and BPH effectors. Despite these advancements, rice pest management faces significant challenges. First, most BPH resistance genes confer only low levels of resistance, limiting their effectiveness. Second, the resistance genes originate from diverse geographical and genetic backgrounds, resulting in variable performance due to environmental and genetic factors and complicating breeding efforts. Third, traditional breeding methods rely on time-consuming hybridization and backcrossing, and introducing resistance genes without affecting other agronomic traits is critical. Fourth, dominance relationships affect resistance levels; heterozygous offspring often exhibit significantly weaker resistance compared to homozygous types [44]. Fifth, while research on BPH resistance mechanisms is extensive, there is a lack of in-depth exploration of the interactions between resistance genes and BPH effectors. To tackle these challenges, China has developed and approved new BPH-resistant rice varieties using traditional breeding combined with molecular marker-assisted selection. “Keguiyou 4302” is the first approved high-quality rice variety resistant to blast disease, bacterial blight, and BPH. “Chengliangyou 4312”, a hybrid mid-season rice bred by China National Seed Group Co., Ltd., shows moderate resistance to both blast disease and BPH. “Bliangyou 164”, developed by the Sanming Agricultural Science Institute, is a stable, high-yielding quality rice variety with moderate resistance to blast and BPH. The promotion of these new varieties has effectively enhanced disease and pest resistance while improving yield and agronomic traits.

### 8.2. Molecular Mechanisms of BPH Resistance Genes and Rice Defense Strategies

Among the cloned genes for resistance to BPH, only a subset has had their molecular mechanisms thoroughly elucidated. These mechanisms mediate rice resistance to BPH through chemical and physical means. For example, the *Bph14* gene, expressed in vascular tissues, activates the SA signaling pathway and induces callose deposition in phloem cells and the production of protease inhibitors upon BPH infection, thus inhibiting the feeding, growth rate, and lifespan of BPH [38]. The *Bph6* gene encodes a protein of the LRD domain family located in the extracellular exosome, which activates the cytokinin, SA, and JA signaling pathways. Interaction with the exosome subunit OsEXO70E1 enhances exocytosis, thereby promoting cell wall stabilization and thickening [45]. The *Bph30* gene, derived from the traditional variety AC-1613, encodes a novel resistance protein containing two LRD domains. It is strongly expressed in sclerenchyma cells, promoting the synthesis of cellulose and hemicellulose, which toughens and thickens the cell walls [44]. By deeply analyzing these molecular mechanisms of BPH resistance genes, we not only understand how rice resists BPH feeding but also reveal the strategies rice employs using secondary metabolites to combat BPH, such as by increasing sakuranetin content or reducing serotonin levels to influence resistance levels [89,148]. These secondary metabolites might also attract natural enemies of BPH, like the egg parasitoid wasp, facilitating oviposition and parasitism and thus forming a biological control in the natural environment. These detailed analyses of molecular mechanisms provide valuable references for rice breeding, enabling breeders to more purposefully create rice varieties with specific resistances. Moreover, in practical production, integrating measures such as crop rotation between wet and dry fields, intercropping, and the judicious use of pesticides can also effectively control BPH populations, reducing their threat to rice production. This integrated management strategy will enhance the enduring resistance of rice and improve the sustainability of agricultural production.

### 8.3. Research on Salivary Proteins of BPH and the Mechanism of Insect Resistance

BPH saliva plays a crucial role in its feeding and digestion processes, helping to dissolve food and produce digestive enzymes for nutrient absorption. Previous studies have revealed the salivary proteome of BPH through various methods, such as transcriptome sequencing of salivary glands from BPH populations with different virulence levels [112], combined LC-MS/MS proteomics and analysis of genomic and transcriptomic data to examine gelled and aqueous saliva isolated from BPH on artificial diets [114], and the use of LC-MS/MS proteomics techniques alongside BPH salivary gland transcriptome databases to identify salivary proteins from BPH-infested feed [113]. These studies indicate that the number of identified salivary proteins varies due to differences in sequencing technologies, BPH populations, and sampling sites. Salivary proteins mainly include salivary phenol oxidase, α-glucosidase, β-glucosidase, oxidoreductases, hydrolases, phosphatases, peptidases (proteases), kinases, transferases, and lyases. Additionally, ATP-binding proteins, lipid-binding proteins, calcium-binding proteins, myosin-binding proteins, and non-enzymatic proteins such as ubiquitin, heat shock proteins, ribosomal proteins, and immunoglobulins have been identified [113]. Recent research further indicates that the role of BPH saliva should not be limited to protein components. Salivary miRNAs, such as miR-100-5P, miR-7-5P, miR-184-3P, miR-1-3P, and miR-9a-5P, can also be secreted in large amounts into plant tissues, triggering strong insect resistance responses [129]. These findings provide a new perspective on the complex interactions between BPH and rice and are of significant importance for developing new insect resistance strategies.

## Supplementary Materials

The following supporting information can be downloaded at: https://www.mdpi.com/article/10.3390/ijms252413397/s1.
