# Supplementary material for: Insight into Rice Resistance to the Brown Planthopper: Gene Cloning, Functional Analysis, and Breeding Applications
Source: Int J Mol Sci. 2024 Dec 13;25(24):13397. doi: 10.3390/ijms252413397 (PMC11678690; doi:10.3390/ijms252413397)
Supplement: Supplementary file 1 [file ijms-25-13397-s001.zip › Table S1.pdf]

Table S1. Mapping of brown planthopper resistance genes/QTL in rice

Genes/QTLs	Germplasm	Chromosome	Linked Markers	Position (Mbp)	Antibiotype
Bph33(t)	RP2068	1L	RM488-RM11522	24.8-28.0	-
Bph37	IR64	1L	RM302-YM35	19.10-19.20	-
Bph38(t)	Khazar	1L	SNP693369-id10112165	20.80-20.90	-
Bph13(t)	*O. eichingeri*	2L	RM240-RM250	31.50-32.78	1,2
Bph13(t)	IR54745-2-21(*O.officinalis*)	3S	AJ09b-AJ09c	5.18-5.70	4
bph19(t)	AS20-1	3S	RM6308-RM3134	7.18-7.24	2
qBph3	IR02W101 (*O.officinalis*)	3L	t6-f3	25.63-35.47	-
bph11	*O. officinalis*	3L	G1318	35.60-35.80	1,2

Bph14	B5 (*O. officinalis*)	3L	SM1, G1318	35.68-35.70	1,2,3
Bph31	CR2711-76	3L	PA26-RM2334	26.25-26.57	4
Bph3	Rathu Heenati	4S	RHD9-RHC10	6.20-6.97	1,2,3,4
qBph4	IR02W101 (*O.officinalis*)	4S	p17, xc4-27	6.70-6.90	-
qBph4	BP360e	4S	RM16382-INDEL4-5	6.20~6.70	-
qBph4.1	Rathu Heenati	4S	-	5.78-7.78	-
qBph4.2	IR65482-17-511 (*O. officinalis*)	4S	RM261-XC4-27	6.58~6.89	-
qBph4.3	Salkathi	4S	RM551-RM335	0.17-0.68	-
qBph4.4	Salkathi	4S	RM335-RM5633	0.68-13.07	-
Bph12	B14(*O. latifolia*)	4S	RM16459-RM1305	5.21-5.56	-

Bph12(t)	*O. latifolia*	4S	RM261-RM8213	4.44-6.57	1,2,3
Bph15	B5 (*O. officinalis*)	4S	RG1-RG2	6.68-6.90	1,2,3
Bph17	Rathu Heenati	4S	RM8213-RM5953	4.44-9.38	1,2
Bph20(t)	*O. minuta*	4S	B42-B44	8.76	1
Bph30	AC-1613	4S	SSR28-SSR69	0.92-0.94	1,2,3,4
Bph33	Kolayal, Poliyal	4S	H99-H101	0.91-0.97	-
Bph35	RBPH660 (*O.rufipogon*)	4S	RM3471-PSM20	6.28-6.94	-
Bph36	GX2183 (*O. rufipogon*)	4S	S13-X48	6.46~6.50	1,2
Bph40	SE232, SE67, C334	4S	-	4.48-4.49	1,2,3
Bph41	SWD10	4S	SWRm_01617-SWRm_01522	0.90-1.10	-

Bph41	GXU202 (*O. rufipogon*)	4S	W4_4_3-W1_6_3	4.68~4.78	-
bph42	*O. rufipogon*	4S	RM16282-RM16335	9.07-9.58	4
Bph44(t)	IRGC 15344	4S	344-0-6, 344-1-2	0.94-1.00	-
qBph4.2	Rathu Heenati	4L	-	15.22–17.22	-
Bph6	Swarnalata	4L	H-Y9	21.40	4
bph12	*O. officinalis*	4L	G271-R93	20.34-21.31	-
Bph16	*O. officinalis*	4L	G271-R93	20.17-21.14	1,2
bph18(t)	*O. rufipogon*	4L	RM273-RM6506	24.05-25.05	2
Bph22(t)	*O. glaberrima*	4L	RM471-RM5742	18.99-21.56	-
bph22(t)	*O. rufipogon*	4L	RM8212-RM261	19.11-19.57	-
Bph27	GX2183(*O. rufipogon)*	4L	RM16846-RM16888	19.12~19.50	2

Bph27(t)	Balamawee	4L	Q52-Q20	20.79-21.33	-
Bph34	*O. nivara*	4L	RM16994-RM17007	21.23-21.32	-
Bph38	*GX2183(O. rufipogon)*	4L	YM112-YM190	15.00-15.10	1,2
Bph42	*SWD10*	4L	SWRm_01695-SWRm_00328	20.60-21.80	–
Bph44	Balamawee	4L	Q31-RM17007	21.38-21.47	-
Bph45	Tainung71 (*O. nivara*)	4L	—	13.70-13.80	-
Bph3	Ptb33,Rathu Heenati	6S	RM589-RM588	1.38~1.61	1,2,3,4
bph4	Babawee	6S	RM589-RM586	1.38-1.47	1,2,3,4
qBph6	IR71033-121-15	6S	RM469-RM568	5.64~5.71	-
bph20(t)	*O. rufipogon*	6S	BYL7-BYL8	0.47-0.53	1

Bph25	ADR52	6S	S00310-RM8101	0.21	-
bph29	*O. rufipogon*	6S	BYL8-BID2	0.48-0.49	1,2
Bph32	Ptb33	6S	RM19291-RM8072	1.21-1.40	1,2
Bph37	SE382	6S	-	1.20-1.57	-
Bph39	Paedai Kalibungga	6S	I7494-I1540	1.07-1.15	-
bph8(t)	Col.5, Col.11,Chin saba	6	RM510-RM314	2.83-4.84	-
qbph8	Swarnalata	8L	RM339-RM515	17.94~20.28	-
bph23(t)	*O. rufipogon*	8L	RM2655-RM3572	16.63-17.07	-
bph21(t)	*O. rufipogon*	10S	RM222-RM244	2.62-5.00	1
qbph11	DV85	11L	XNpb202-C1172	17.43~19.56	-
Bph28(t)	DV85	11L	Indel55-Indel66	16.90-16.96	2

Bph43	IRGC 8678	11L	InDel16_22-InDel16-30	16.79~16.90	-
qBph11.3	CL48	11L	11M16.781-11M16.896	16.74-16.89	-
Bph1	Mudgo	12L	pBPH4-pBPH14	22.86	1,3
bph2	ASD7	12L	RM463-RM7102	22.87-22.89	1,2
Bph7	T12	12L	RM3448-RM313	19.95-20.87	1,2,3,4
Bph9	Pokkali	12L	InD2-RsaI	22.85-22.97	1,2,3
Bph9	Kaharamana	12L	RM463-RM5341	22.87-	1,2,3
Bph10	*O. australiensis*	12L	RG457	19.55-26.98	1,3
qBph12	ASD7	12L	RM28466-RM7376	22.94~23.44	
Bph18	IR65482-7-216-1-2 *(O. australiensis)*	12L	BIM3-BN162	22.88	1,3

bph19(t)	*O. rufipogon*	12L	RM17	26.98	2,九龙江型
Bph21	IR71033-121-15 (*O. Minuta)*	12L	S12094A-B122	24.20-24.36	1,3
Bph26	ADR52	12L	DS72B4-DS173B	22.87-22.89	1,2
Bph46	CL45	12L	SNP-5-SNP-6	18.15-18.61	-
bph5	ARC10550	-	-	-	4
Bph23(t)	*O. minuta*	-	-	-	-
bph24(t)	IR73678-6-9-B (*O.rufipogon*)	-	-	-	-
bph39(*t*)	*O. nivara*	-	-	-	-
bph40(*t*)	*O. nivara*	-	-	-	-
