# Supplementary material for: Insight into Rice Resistance to the Brown Planthopper: Gene Cloning, Functional Analysis, and Breeding Applications
Source: Int J Mol Sci. 2024 Dec 13;25(24):13397. doi: 10.3390/ijms252413397 (PMC11678690; doi:10.3390/ijms252413397)
Supplement: Supplementary file 1 [file ijms-25-13397-s001.zip › Table S2.pdf]

Table 2. Cloning of rice brown planthopper resistance genes

Gene	Germplasm	Chromosome	Encoded Protein	Subcellular Localization	Resistance	Year
Bph14	B5 (*O. officinalis*)	3L	CC-NB-LRR	cytoplasm and Nucleus	BPH	2009
Bph15	B5 (*O. officinalis*)	4S	Lectin receptor kinase	Plasma membrane	BPH	2013
Bph3	Rathu Heenati	4S	Lectin receptor kinases	Plasma membrane	BPH、WBPH	2015
Bph30	AC-1613	4S	LRD	Endomembrane system	BPH、WBPH	2021
Bph40	SE232, SE67, C334	4S	LRD	-	BPH	2021
Bph6	Swarnalata	4L	LRD	Exocyst	BPH、WBPH	2018
bph29	RBPH54	6S	B3 DNA-binding	Nucleus	BPH	2015
Bph32	Ptb33	6S	SCR	Plasma membrane	BPH	2016
Bph37	SE382	6S	CC-NB	-	BPH	2021

Bph1	Mudgo	12L	CC-NB-NB-LRR	Endomembrane system	BPH	2016
Bph2	ASD7	12L	CC-NB-NB-LRR	Endomembrane system	BPH	2016
Bph7	T12	12L	CC-NB-NB-LRR	Endomembrane system	BPH	2016
Bph9	Pokkali	12L	CC-NB-NB-LRR	Endomembrane system	BPH	2016
Bph10	IR65482-4-136-2-2 *(O. australiensis)*	12L	CC-NB-NB-LRR	Endomembrane system	BPH	2016
Bph18	IR65482-7-216-1-2 *(O. australiensis)*	12L	CC-NB-NB-LRR	Endomembrane system	BPH	2016
Bph21	IR71033-121-15 (*O. minuta*)	12L	CC-NB-NB-LRR	Endomembrane system	BPH	2016
Bph26	ADR52	12L	CC-NB-NB-LRR	Endomembrane system	BPH	2014
